# Supplementary figures and images for: The LysR-Type Transcriptional Regulator BsrA (PA2121) Controls Vital Metabolic Pathways in Pseudomonas aeruginosa
Source: mSystems. 2021 Jul 13;6(4):e00015-21. doi: 10.1128/mSystems.00015-21 (PMC8407307; doi:10.1128/mSystems.00015-21)

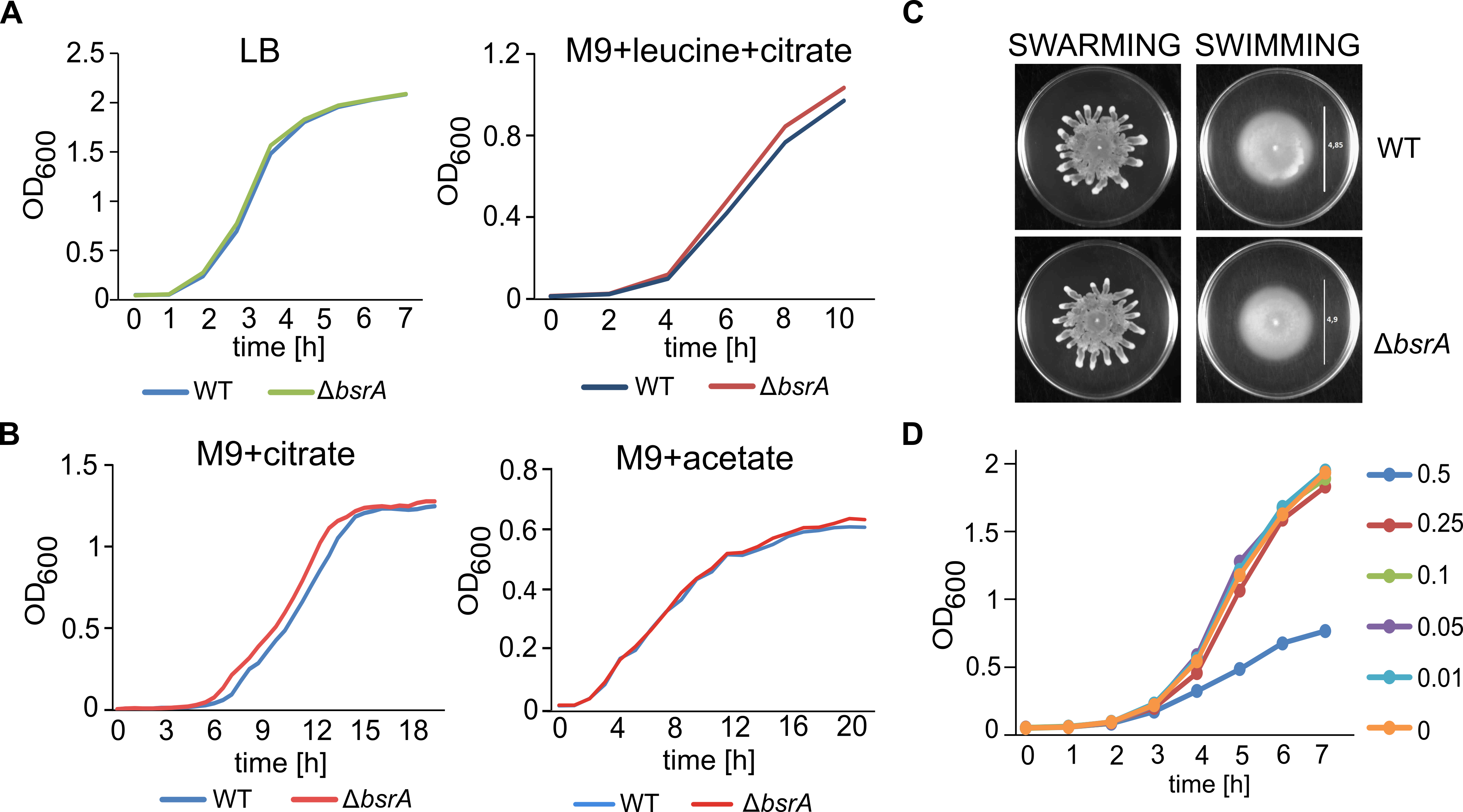

Supplement: FIG S1 [file msystems.00015-21-sf001.tif]

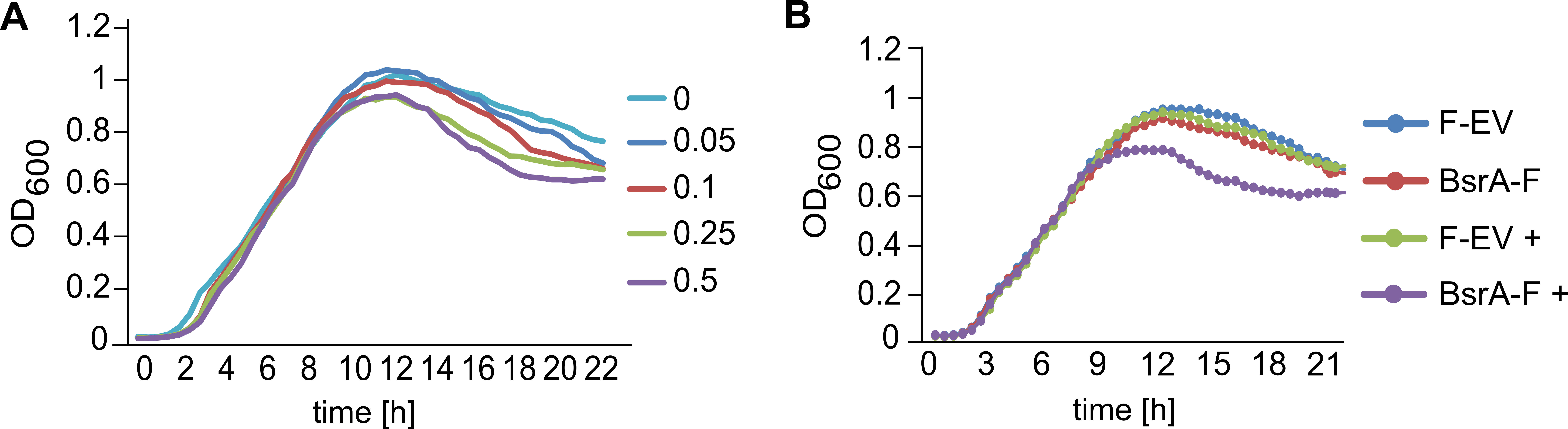

Supplement: FIG S2 [file msystems.00015-21-sf002.tif]
